# Are Alzheimer's and coronary artery diseases genetically related to longevity?

**DOI:** 10.3389/fpsyt.2022.1102347

**Published:** 2023-01-06

**Authors:** Eftychia Bellou, Valentina Escott-Price

**Affiliations:** ^1^UK Dementia Research Institute, School of Medicine, Cardiff University, Cardiff, United Kingdom; ^2^Division of Neuroscience and Mental Health, School of Medicine, Cardiff University, Cardiff, United Kingdom

**Keywords:** pleiotropy, Alzheimer's disease, coronary artery disease, longevity, subset-based analysis (ASSET)

## Abstract

**Introduction:**

In the last decade researchers have attempted to investigate the shared genetic architecture of longevity and age-related diseases and assess whether the increased longevity in certain people is due to protective alleles in the risk genes for a particular condition or whether there are specific “longevity” genes increasing the lifespan independently of age-related conditions' risk genes. The aim of this study was to investigate the shared genetic component between longevity and two age-related conditions.

**Methods:**

We performed a cross-trait meta-analysis of publicly available genome-wide data for Alzheimer's disease, coronary artery disease and longevity using a subset-based approach provided by the R package ASSET.

**Results:**

Despite the lack of strong genetic correlation between longevity and the two diseases, we identified 38 genome-wide significant lead SNPs across 22 independent genomic loci. Of them 6 were found to be potentially shared among the three traits mapping to genes including *DAB2IP, DNM2, FCHO1, CLPTM1*, and *SNRPD2*. We also identified 19 novel genome-wide associations for the individual traits in this study. Functional annotations and biological pathway enrichment analyses suggested that pleiotropic variants are involved in clathrin-mediated endocytosis and plasma lipoprotein and neurotransmitter clearance processes.

**Discussion:**

In summary, we have been able to advance in the knowledge of the genetic overlap existing among longevity and the two most common age-related disorders.

## 1. Introduction

The average human life expectancy has increased in the last decades, but this has not been accompanied by a similar increase in health span. Aging is the driving factor of various age-related diseases (ARDs), including Alzheimer's disease (AD) and cardiovascular disorders, causing a significant burden on social and economic level. Despite the well-known importance of age in our understanding of diverse diseases, the molecular mechanisms by which aging exerts these effects are still mostly unknown (1).

It has been suggested that aging and ARDs are part of a continuum with the two extremes being (a) individuals who suffered one or more ARDs at the age of 60 or 70 years and show signs of accelerating aging, and (b) centenarians who avoided ARDs or postponed their onset. Whether an individual will manifest an ARD and follow a trajectory of accelerated or decelerating aging depends on factors such as the genetic background of the individual, their lifestyle, and various environmental conditions (2). This concept fits well with the idea of hormesis in aging in which lifelong low-intensity exogenous factors/stressors stimulate maintenance and repair mechanisms with beneficial effects for health, whereas the increase of the intensity can overcome the capability of the organism to adapt and end up with detrimental effects such ARDs (3, 4).

Meanwhile, studies in older-aged cohorts and specifically in centenarians have shown that the latter do not achieve exceptional longevity due to the absence of variants that predispose them to the risk of mortality-leading diseases, but rather it seems that are enriched with protective genes related to a reduced risk of e.g., cardiovascular diseases and metabolic disorders (5). The genetic buffering mechanism has been introduced in which many longevity genes, which are enriched in centenarians, have the ability to buffer against the harmful effects of deleterious genotypes *via* gene-gene interactions (6, 7). In the last decade there were attempts to study the shared genetic architecture of longevity and ARDs to assess the latter hypothesis and whether the increased longevity in certain people is due to protective alleles in the risk genes for a particular condition or whether there are specific “longevity” genes increasing the lifespan independently of age-related conditions' risk genes. Sebastiani and co-authors (8) showed that 130 genes associated with the human lifespan were enriched for several groups of genes linked to both AD and Coronary Artery Disease (CAD) including *TOMM40/APOE* and *CDKN2A*. On the contrary, in study by Beekman et al. (9) the authors compared the cumulative effect of risk alleles for cardiovascular disease, type 2 diabetes, and cancer between individuals aged 85 years and older and the middle-aged general population and found that longevity is not compromised by this cumulative effect. This could be due to an increased prevalence of protective alleles for the diseases under study that could lead to the delay of disease onset or to the decrease of the severity. Tesi et al. (10) explored the genetic architecture between AD and longevity by studying the effect of 38 AD-associated genetics variants on longevity, and they showed that 74% of the AD risk alleles are associated with lower odds of becoming a centenarian. Finally, in a recent study by Martin and Fraser (11) the authors suggested that part of the pathogenesis of the ARDs might be partly regulated by loci that control gene expression over age. They managed to identify genetic variants in the human brain that control messenger RNA (mRNA), DNA methylation and microRNA (miRNA) levels in an age-dependent manner.

In this study we aimed to utilize a pleiotropic meta-analytic approach to comprehensively parse variance from AD, CAD, and longevity focused genome-wide association studies (GWASs) that might pinpoint differential biological mechanisms and aid in understanding the effect of these age-related diseases' variants on longevity. These diseases were chosen as they are most prevalent (and therefore best studied) diseases (12, 13) representing neurodegenerative and cardiovascular conditions in the population, and age is their strongest factor related to their development and mortality. Moreover, growing literature indicates that cardiovascular disease risk factors, including high blood pressure, high low-density lipoprotein cholesterol, diabetes and obesity are associated with increased risk of AD, and its precursor, cognitive decline (14). Finally, the current study could shed some light into the validity of the cardiovascular dementia hypothesis (15) in which aortic stiffness, a key indicator of cardiovascular diseases, may result in brain damage as well as heart failure due to the relation of proximal aorta with both the heart and brain perfusion. Thus, the understanding of a potential vascular etiology in AD could have an important role in developing effective preventive strategies.

## 2. Material and methods

### 2.1. Samples and summary statistics data

For the current analysis, we used publicly available GWAS summary statistics for the three traits. For AD we used the stage one meta-analysis data (*N* = 63,926) from Kunkle et al. (16), for CAD the data from the CARDIoGRAMplusC4D 1,000 Genomes-based GWAS (*N* = 184,305) by Nikpay et al. (17), and finally, for longevity we used the meta-analysis data corresponding to the 90th survival percentile (*N* = 36,745) by Deelen et al. (18). Detailed information about study designs, quality-control and meta-analytic procedures were reported by the authors to each publication, respectively. All three datasets were harmonized to the same allele using as a reference the 1,000 Genomes Project Phase 3 data (19). Multi-allelic and ambiguous genetic variants were removed from the datasets along with variants with minor allele frequency (MAF) < 0.01 as in 1,000 genomes. Moreover, as the mean imputation information was available for CAD, thus we additionally removed variants with imputation quality score INFO < 0.75 in that dataset. It should be noted that the summary statistics data used for the analyses are not taken from the largest, most recent GWASs; rather, we used smaller GWASs that do not include the UK Biobank. We, however verified our findings against the more recent studies that have been published for AD (20–22), longevity (23) and CAD (24).

### 2.2. Estimation of the genetic correlation among the traits

The genetic correlation (*r*_g_) describes the genetic similarity of two traits by capturing the extent to which genetic factors influence their covariance. The genetic correlation is expected to reflect the existence of pleiotropy; however, these two concepts do not have the same meaning (25). For example, an estimation of *r*_g_ close to zero does not necessarily imply that the two phenotypes do not share risk loci. Thus, to capture the latter scenario we estimated both the whole-genome and the local correlation between the three traits.

#### 2.2.1. Whole-genome correlations

Cross-trait LD score regression (LDSC) (26) (https://github.com/bulik/ldsc) was used to test the genetic overlap among each pair of traits using the HapMap3 (27) variants as proposed by the developers. The key assumption behind this method is that the variants with a high LD score - a measure of the extend of the linkage disequilibrium (LD) between a variant and its neighbor variants - are more likely to tag a causal single nucleotide polymorphism (SNP) and have a higher χ^2^ statistics than SNPs in a low-LD region (28). The analysis was performed using the pre-computed European SNP LD scores (https://data.broadinstitute.org/alkesgroup/LDSCORE/eur_w_ld_chr.tar.bz2) and the major histocompatibility region (MHC) was excluded as recommended.

#### 2.2.2. Local correlations

The LAVA (Local Analysis of [co]Variant association) (29) (https://github.com/josefin-werme/LAVA) was performed to identify the pairwise local genetic correlations among the three phenotypes. Since the LDSC only considers the average *r*_g_ across the entire genome, LAVA approach can detect scenarios where the signal is confined to specific regions or in opposing directions at different loci (25). The LAVA analysis used the genetic covariance intercept from the LDSC analyses to adjust for potential sample overlap and the local *r*_g_ was tested within the 2,495 genome-wide loci as constructed and described by Werme et al. (29). Initially, a univariate analysis for each trait at each locus was performed to ensure that sufficient local heritability was present to perform bivariate local r_g_ analysis. Then for each trait pair, the bivariate analysis was performed only for loci in which both phenotypes exhibited univariate signal at *p* < 0.05/2,495 resulting in 16 bivariate tests conducted in total. Bivariate analyses results were considered significant when *p* < 0.05/16. To account for potential samples overlap, LAVA requires the covariance intercept from LDSC analysis. Furthermore, partial correlation analysis was performed with LAVA to examine any conditional genetic relationships in more detail for the LD blocks with significant local *r*_g_.

### 2.3. Association analysis based on subsets

We applied a subset-based meta-analysis (30) using the R package ASSET to identify pleiotropic SNPs. The approach is specifically designed for detecting association signals across multiple phenotypes accounting for subset-specific and bidirectional effects of individual variants. ASSET searches across all possible subsets of the input GWAS traits to identify the strongest association signal in both positive and negative directions and returns a *p*-value (multiple-testing corrected) for the overall evidence of association of a variant across phenotypes along with the best subset of phenotypes that contributed to the overall association. The method also allows to account for potential sample overlap (30).

We combined the GWAS summary statistics from AD, longevity and CAD by using ASSET function “h.traits” with default parameters to perform a two-sided search to obtain pleiotropic variants that may be associated with different phenotypes in different directions of the association. Only SNPs that were present for all three traits were retained as inputs to the meta-analysis, resulting in 5,987,749 SNPs. As above, inter-study correlations from LDSC were used to account for sample overlap. After subset-based meta-analysis, SNPs were considered statistically significant when (1) their ASSET-derived overall *p*-value was lower than 5e-08, (2) both *p*-values for the positively and negatively associated subsets of traits were lower than 0.05, and (3) the *p*-values from the initial GWASs input studies reached at least nominal significance (*p* < 0.05).

#### 2.3.1. Consolidation of independent loci and functional annotation

Independent loci in the ASSET-derived results were identified *via* LD-clumping using SNP2GENE pipeline from the online platform FUMA (https://fuma.ctglab.nl) (31) on the basis of the European 1,000 Genomes Project phase 3 reference panel (19). Initially, independent significant SNPs with *p*-value < 5e-08 and independent from each other at (*r*^2^ < 0.6) were identified. Then, candidate SNPs, defined as all known SNPs of with *p*-value < 0.05 and *r*^2^ ≥ 0.6 with one of the independent significant SNPs, were identified for further annotations. Finally, based on the candidate SNPs independent lead SNPs were defined as the SNPs with the strongest association at a given locus and with *r*^2^ < 0.1 from each other. Genomic risk loci that were 250 kb or closer to each other were merged into one locus.

Functional consequences of the candidate SNPs in each risk loci were obtained by performing ANNOVAR (32) using Ensembl genes as described by FUMA's developers (31). In summary, these SNPs were first matched based on chromosome, base pair position and reference allele to a database containing functional annotations including the ANNOVAR categories (32), combined annotation dependent depletion (CADD) scores (33) and Regulome DB (RDB) scores (34). The ANNOVAR categories were used to identify the function of the SNP, and to locate its position within the genome (positional mapping). CADD scores are a measurement used to determine how deleterious genetic variation at the SNP is to protein structure and function. Higher scores are indicative of a more deleterious variant, with scores of >12.37 providing evidence of pathogenicity (33). A Regulome DB score is a categorical measurement based on data from expression quantitative trait loci (eQTLs) as well as chromatin marks. The RDB score ranges from 1a to 7 with lower scores given to the variants with the greatest evidence for having regulatory function.

#### 2.3.2. Gene mapping and identification of novel individual-trait associations

The functionally annotated SNPS were mapped in genomic risk loci using two approaches: (a) positional mapping in which SNPs are physically located within protein-coding genes (10 kb windows are used) and (b) eQTL mapping in which all independent significant SNPs and their proxies were mapped to genes based on a significant eQTL association, by using information from four data repositories; GTEx v8 (35) tissues (Blood, Blood vessel, Brain, Heart), PsyENCODE (http://resource.psychencode.org) (36), DICE (https://dice-database.org) (37) and BRAINEAC (http://www.braineac.org/) (38). By default, a false discovery rate (FDR) of 0.05 was applied to define significant eQTL associations, and SNPs were mapped to genes up to 1 Mb apart. MHC region was excluded from annotations.

Finally, to identify individual trait novel associations, we investigated whether the pleiotropic SNPs identified by ASSET were associated at genome-wide level with any of the input traits (and more) using GWAS Catalog (https://www.ebi.ac.uk/gwas/).

#### 2.3.3. Gene-based and gene-set analysis using MAGMA

Both gene analysis and gene-set analysis were performed using MAGMA (39) by the FUMA pipeline as described in (31). In summary, for the SNP-wise gene-based analysis, SNPs were mapped to protein-coding genes (gene window: 0 kb) and the gene-based *p*-value was estimated. For the gene set analysis under the competitive model, the gene set *p*-value was computed using the gene-based *p*-value for 4,728 curated gene sets (including canonical pathways) and 6,166 GO terms obtained from MsigDB v5.2 (40). Results were Bonferroni multiple testing corrected.

#### 2.3.4. Enrichment analysis of the mapped genes in pre-defined pathways

To obtain insight into putative biological mechanisms of the mapped genes (based on functional and eQTL mapping), we used the GENE2FUNC process implemented on the online platform FUMA to annotate these genes in biological context. We conducted enrichment analysis of the genes against gene sets from biological pathways and functional categories obtained from MsigDB (40) and WikiPathways (41).

## 3. Results

### 3.1. Genetic correlations

Consistent with previous studies (18, 42, 43), LDSC returned negative genome-wide genetic correlations among the three traits: AD-longevity *r*_g_ = −0.18 (*p* = 0.16), AD-CAD *r*_g_ = −0.11 (*p* = 0.11), CAD-longevity *r*_g_ = −0.39 (*p* = 9.82e-09), with the genetic correlation between CAD and longevity being the only significant one.

LAVA analysis revealed only one significant local r_g_ between all traits at chr19:45,040,933–45,893,307. This region contains the well-known *APOE* gene with which all three traits present a strong association. As expected, the correlation of AD and CAD traits with longevity was found to be negative (AD-longevity local *r*_g_ = −0.79 and *p* = 3.49e-55, CAD-longevity local *r*_g_ = −0.71 and *p* = 1.34e-11), whereas the *r*_g_ between AD and CAD in the region was found to be positive (*r*_g_ = 0.54, *p* = 1.18e-12). Notably, only 1 out of 15 loci with significant univariate local heritability for at least two phenotypes had a bivariate significant *p*-value, suggesting that strong local heritability can occur in the absence of any local *r*_g_.

To further test the relationship at chromosome 19 region, we performed conditional analysis using partial correlation to determine whether any component of the r_g_ between longevity and AD remained once accounting for CAD and vice versa. Conditioning on CAD, the partial correlation between longevity and AD remained high (partial *r*_g_ = −0.69, *p* = 5.87e-04 vs. bivariate *r*_g_ = −0.79). Similarly conditioning on AD, a sizeable portion of the original association between longevity and CAD remained high (partial *r*_g_ = −0.55, *p* = 3.62e-04 vs. bivariate r_g_ = −0.71). These results indicate that the local *r*_g_ between each pair of traits is not driven by the third trait and thus, the local genetic signals for both AD and CAD confer a greater chance of not reaching the 90th survival percentile.

### 3.2. Association analysis based on subsets

#### 3.2.1. Identification of independent loci and functional annotations

**Figure 1:**
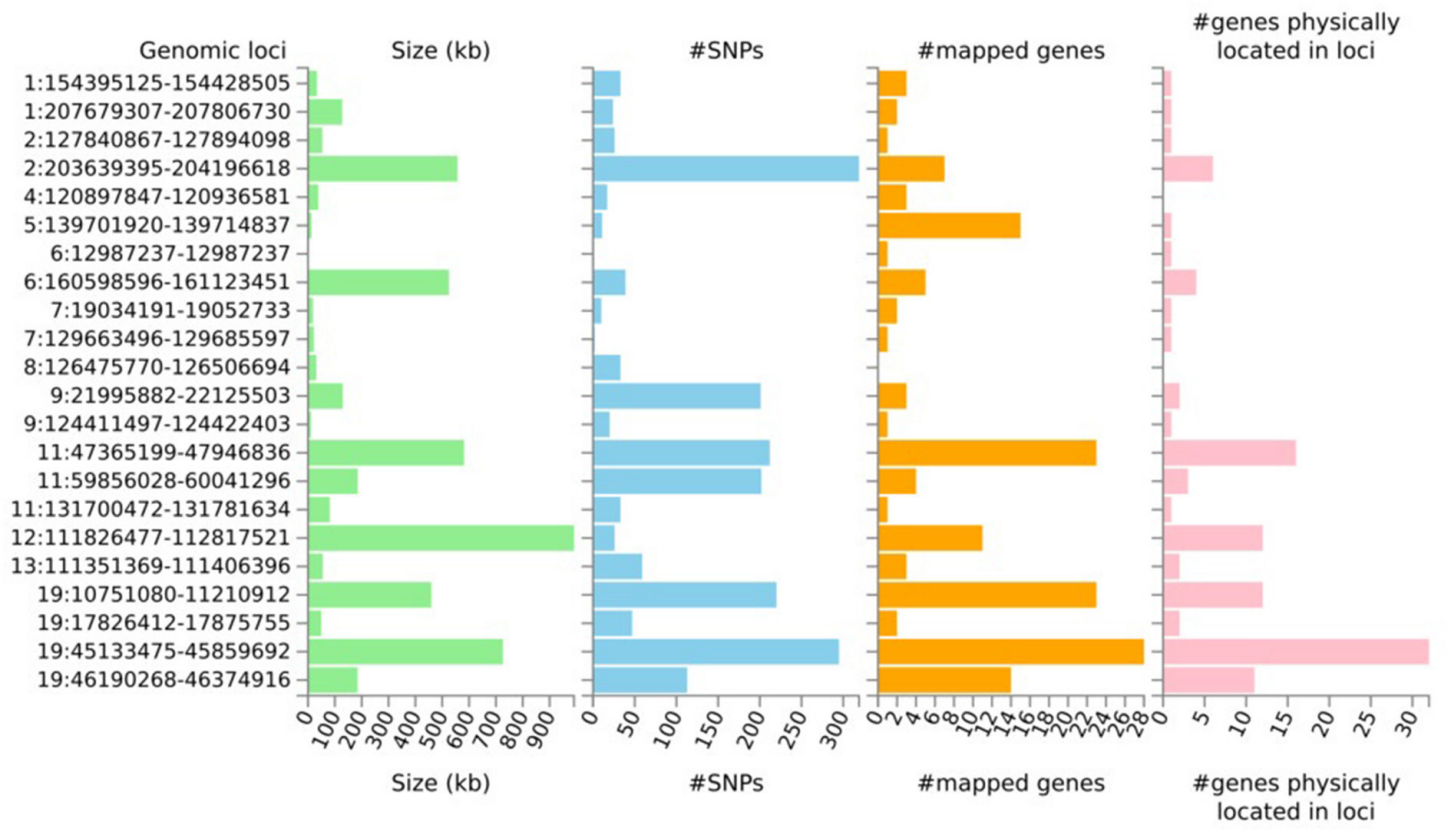
Summary results per genomic locus identified from the subset-based meta-analysis (ASSET) after LD clumping. The histograms display the size of genomic loci, the number of both candidate SNPs and mapped genes, and the number of genes physically located within the locus.

Despite the lack of strong genetic correlation between longevity and the two age-related diseases, we performed the two-sided ASSET cross-trait analysis with the intention to boost the power to detect loci shared by at least two of the three phenotypes. When doing so, the meta-analysis revealed 10,662 SNPs with nominally significant *p*-values (*p* < 0.05) for both the positively and negatively associated subsets and the initial GWAS input studies. After LD-clumping 38 lead SNPs across 22 independent genomic loci (see Figure 1, Supplementary Figure S1, and Supplementary Tables S1–S3) were identified. The three top lead SNPs were rs34095326 in the intronic region of *TOMM40* (p_ASSET_ = 0), rs2891168, a non-protein-coding RNA at chromosome 9 (p_ASSET_ = 1.40e-98, *CDKN2B-AS1*) and rs118039278 in the intronic region of *LPA* (p_ASSET_ = 4.38e-37) (Supplementary Tables S3, S4). Both rs2891168 and rs118039278 were identified as pleiotropic for longevity and CAD, whereas rs34095326 was shared between AD and longevity.

Four of the 22 genomic risk loci (or 6/38 independent lead SNPs) were flagged as pleiotropic for the all the three traits; two SNPs were included in the clusters Longevity|AD, CAD and surprisingly, four in the cluster CAD|AD, Longevity (Supplementary Table S3). More specifically, the two SNPs located in chromosome 19 (82 Kb and 34 Mb away from the *APOE* gene, respectively) were positively associated with longevity and negatively associated with AD and CAD: rs117261169-T OR_cluster_ = 1.39, P_cluster_ = 1.20e-04 vs. OR_cluster_ = 0.78, P_cluster_ = 8.85e-09, and rs2043332-A OR_cluster_ = 1.05, P_cluster_ = 2.39e-02 vs. OR_cluster_ = 0.95, P_cluster_ = 2.48e-08, respectively. In other words, both alleles are protective of developing AD and CAD leading to higher chances of reaching the 90th percentile of aging. Interestingly, the G allele of rs10774624 (locus 17, *RP3-473L9.4*), rs1964272 (locus 22, *SNRPD2*), rs10818576 (locus 13, *DAB2IP*), and rs9630903 (locus 20, *FCHO1*) has the same direction of association for AD and longevity and opposite for CAD despite the negative genetic correlation between AD and longevity (Supplementary Tables S3, S4).

In the pair-wise analysis between AD and longevity, 18 independent lead SNPs from 7 risk loci showed a pleiotropic opposite effect with most of them being in the *APOE*/*TOMM40* region (locus 21), verifying the local genetic correlation that was reported in the previous section (Supplementary Table S3). The rest of the SNPs were positionally mapped to *CR1* (rs2093761, locus 2), *BIN1* (rs6733839, locus 3), *HBEGF* (rs11168036, locus 6), *SPI1* (rs67472071, locus 14), *AP001257.1* (rs583296, locus 15), and *NTM* (rs9787911, locus 16).

Pair-wise analysis of longevity and CAD found 11 lead SNPs from 10 genomic loci with opposite direction of effect. SNPs including rs118039278 and rs9457927 (locus 8), rs2107595 (locus 9), rs11556924 (locus 10), rs2980853 (locus 11), rs2891168 (locus 12), and rs56289821 (locus 19) have also been reported to have pleiotropic effects on other cardiovascular diseases and their risk factors such as myocardial infraction, systolic blood pressure, hypertension, cholesterol levels and triglycerides (Supplementary Table S5). Finally, two SNPs showed opposite, pleiotropic effects between CAD and AD; both the rs116426890-T (locus 4) and rs62118504-G (locus 21) were found to increase the risk of CAD (OR = 1.13, *P* = 2.95e-13 and OR = 1.03, *P* = 2.26e-03, respectively) and decrease the risk of AD (OR = 0.95, *P* = 3.46e-02 and OR = 0.89, *P* = 1.06e-11, respectively).

**Figure 2:**
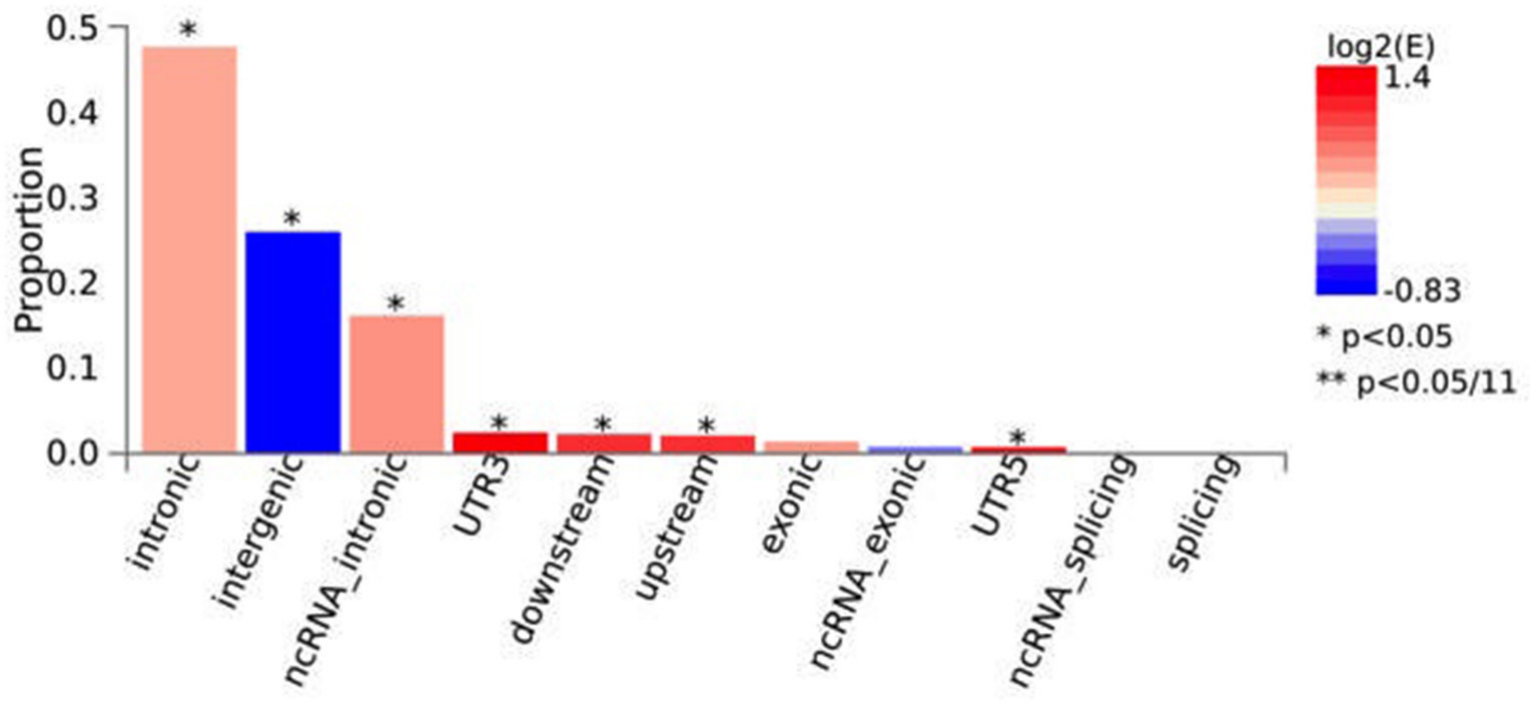
Functional consequences of ASSET candidate SNPs on genes. The histograms display the proportion of SNPs (all SNPs in LD with independent significant SNPs) which have corresponding functional annotation assigned by ANNOVAR. Bars are colored by log2 (enrichment) relative to all SNPs in the selected reference panel. Enrichment is computed as (proportion of SNPs with an annotation)/(proportion of SNPs with an annotation relative to all available SNPs in the reference panel). Proportion is the fraction of candidate SNPs with the corresponding annotation. Fisher's exact test (two side) is performed for each annotation. *p < 0.05; **p < 0.05/11.

Functional annotation conducted in FUMA indicated that, across the independent genomic loci found by ASSET analysis, there was a significant overrepresentation of SNPs found in introns (48%), ncRNA intronic regions (16%), UTR3 (2%), downstream (2%), upstream and in UTR5 regions (0.7%) (Figure 2). In intergenic regions the number of SNPs (26%) were significantly underrepresented. Moreover, 110/1,943 (5.66%) of the candidate SNPs had a Regulome DB Score < 3, indicating that variation at these SNPs is likely to affect gene expression (Supplementary Table S4). Finally, 65/1,943 (3.35%) and 3.79% had a CADD score of >12.37 indicating that variation at these SNPs is deleterious (Supplementary Table S4).

#### 3.2.2. Identification of novel associations

We assessed whether our meta-analysis identified loci have been discovered in more recent and larger GWASs that have been published for AD (20–22), longevity (23) and CAD (24). Of the 22 shared risk loci identified, 11 have already been reported as risk factors for the diseases contributing to the association, according to GWAS Catalog, whereas the rest could potentially be novel loci for at least one of the traits (Supplementary Table S8). Our subset-based meta-analysis indicated locus 4 (lead SNP rs116426890, p_ASSET_ = 3.40e-13, *ABI2*), locus 13 (lead SNP rs10818576, p_ASSET_ = 3.49e-09, *DAB2IP*), locus 16 (lead SNP rs9787911, p_ASSET_ = 9.95e-09, *NTM*), locus 20 (lead SNP rs9630903, p_ASSET_ = 2.43e-08, *FCHO1*) and locus 22 (lead SNP rs1964272, p_ASSET_ = 6.62e-10, *SNRPD2*) to be potentially novel for AD with only rs116426890 reaching a nominal significance of *p* = 1.52e-06 in the Bellenguez et al. study (22). Moreover, the lead SNP rs11168036 from locus 6 that was not significant in the input GWAS and was suggested to be shared with longevity in our study, was now found to be associated with AD (p_stage_1+2_ = 7e−09) in a transethnic study (44). Regarding CAD, from the 15 loci that were found to be shared with at least one of the other traits in this study, nine SNPs (rs11723436, rs2980853, rs9583531, rs117261169, rs1964272, rs10818576, rs2043332, rs9630903) were not GWAS significantly associated with the disease input GWAS; however, eight of them including rs62118504 (*EXOC3L2* gene), rs1964272 (*SNRPD2* gene), rs11723436 (RP11-*170N16*.1 gene), rs10818576 (*DAB2IP* gene), rs2980853 (RP11-*136O12*.2 gene), rs2043332 (DNM2 gene), rs9630903 mapped to *FCHO1* and rs9583531 mapped to *ING1*, were found to be significant in a later and larger CAD GWAS study by van der Harst et al. (24), again proving the utility of ASSET in discovering novel risk loci. Finally, 36 SNPs from 21 loci reported by ASSET were associated with longevity with only rs34095326 (locus 21) being significant in the original GWAS. Four risk loci were found to be significant in a larger GWAS by Timmers et al. (23) and another two SNPs were nominally significant including rs11556924 (*p* = 3.3e-07, locus 10) and rs2980853 (*p* = 3.5e-05, locus 11).

#### 3.2.3. MAGMA gene-based and gene-set analysis

A gene-based analysis was conducted using MAGMA which can increase the power to detect significant associations as the signal across many SNPs (all within a gene) is combined (39). Input SNPs were mapped to 1,117 protein-coding genes and only 200 genes survived the Bonferroni correction significance threshold of 4.476e-5 (*P* = 0.05/1,117) (as seen in Supplementary Table S9). The competitive gene-set analysis uses 63 significant GO biological processes, REACTOME and curated gene-sets (detailed results in Supplementary Table S10). Among these gene sets, there were six gene sets involved in the immunity, including the negative regulation of adaptive immune response (*P* = 1.31e-04) and the negative regulation of leukocyte mediated immunity (*P* = 1.32e-04) and 6 involved in oncogenic processes such as the roversi glioma copy number up (*P* = 6.34e-11) and the oncogene induced senescence (*P* = 9.76e-07).

#### 3.2.4. Enrichment analysis of the mapped genes in pre-defined pathways

Using both positional and eQTL mapping in FUMA, we mapped our SNPs into 151 genes as seen on Supplementary Tables S6, S7. Then we performed overrepresentation analysis of the mapped (physically and eQTL positioned) genes in Reactome DB and GO pre-defined pathways created by the FUMA developers (as seen in Supplementary Table S11) and GENE2FUNC tool. The analysis highlighted two interesting biological processes significantly enriched in the shared genes between the three traits specifically those involve/related to endocytosis. These include the clathrin coated endocytic vesicle (P_FDR_ = 0.02) and the clathrin coated endocytic vesicle membrane (P_FDR_ = 0.03) which have been implicated in the pathology of AD (45, 46) and atherosclerosis (47). Among Reactome pre-defined pathways, seven were found to be statistically enriched including the neurotransmitter clearance (P_FDR_ = 0.02), the plasma lipoprotein clearance (P_FDR_ = 0.03) and the plasma lipoprotein assembly remodeling and clearance (P_FDR_ = 0.04).

## 4. Discussion

Through the current cross-trait meta-analysis of two age-related diseases, AD and CAD, and longevity, we have been able to advance in the knowledge of the genetic overlap between the three phenotypes and show that one of the genetic mechanisms for extreme longevity involves the avoidance of certain risk alleles that predispose to common diseases. Specifically, our meta-analysis identified 38 genetic variants from 22 risk loci shared among subsets of the diseases under study, many of which represent new individual-trait genetic risk loci.

Two of the five pleiotropic loci identified by Fortney et al. (48) using data from centenarian cohorts for age-related traits, *TOMM40/APOE* (shared by longevity and AD) and *CDKN2B-AS1* (shared by longevity and CAD), were also returned by our subset-based meta-analysis (the SNPs mapped in these genes in both studies were in LD).

We also found six independent lead SNPs from six loci to be pleiotropic for the all the three traits. Three SNPs, rs9630903-G, rs117261169-T, and rs1964272-G are spanning chromosome 19 and were mapped to *FCHO1, CLPTM1*, and *SNRPD2*, respectively. The SNP rs9630903-G was found to be associated with a reduced risk of CAD in a larger van der Harst et al. (24) study, verifying our association using a smaller CAD GWAS. The rs9630903 was found to potentially affect the expression of both *FCHO1* and *MAP1S* genes by eQTL mapping (Supplementary Table S7). *FCHO1* is involved in endocytosis *via* clathrin coated endocytic vesicle pathways (49) binds *APP* and its missense mutation NM_001161357.1:c.557G > A (rs147599881) approached significance in the cerebrospinal fluid (CSF) biomarker data in a recent analysis of AD-affected cousin pairs selected from high-risk pedigrees (50). *CLPTM1* resides in the same region as *APOC1* and *CEACAM19* in close proximity with *APOE* which is also flagged as a pleiotropic gene associated with the three phenotypes. *CLPTM1* plays a role in the regulation of GABA receptor trafficking from the ER to the plasma membrane, suggesting that *CLPTM1* could regulate inhibitory neurotransmission (51). Two recent transcriptome-wide studies (TWASs) (52, 53) reported *CLPTM1* to be significantly associated with AD in CD14+ monocyte expression data from the Cardiogenics transcriptomics study and in the hippocampal, the putamens and the nucleus putamens tissues, respectively. Moreover, a genome-wide scan for SNPs involved in exceptional longevity identified rs405509, (*r*^2^ = 0.62 with rs117261169), which is a temporal expression eQTL (teQTL) with the gene *CLPTM1* (*P* = 4.8e-03; longevity increasing allele increases expression over age). Furthermore, rs117261169-T has been found to be associated with self-reported high cholesterol (β = −0.03, *P* = 8.61e-17), a known cardiovascular mediator, and self-reported AD/dementia in mothers (in the UK Biobank) (β = −0.01, *p* = 5.05e-06), further solidifying its potential role as a pleiotropic SNP. The rs1964272 SNP, which was found to be associated with CAD (β = 0.03, *P* = 1.31e-08) in a later study by van der Harst et al. (24), was physically mapped 443kb downstream of *SNRPD2* and to both *SNRPD2* and *DNWD* by eQTL mapping. *SNRPD2* has been implicated in the pathogenesis of both MCI and AD, with decreased expression level (54) and also, AD was affected by the gene expression of *SNRPD2* in the hippocampus and putamen (53) proving that the gene is likely to be pleiotropic.

The other three SNPs returned as pleiotropic for all three phenotypes in our analysis were rs10818576 (*DAB2IP*), rs10774624 (*RP3–473L9.4*), and rs2043332 (*DNM2*). The SNP rs10818576 is intronic to *DAB2IP*, shows no significant with the expression of any gene based on the eQTL mapping and its G allele was reported as decreasing the risk of developed of CAD (24). *DAB2IP* acts as a negative regulator of vascular endothelial growth factor signaling and angiogenesis (55), a tumor suppressor (56) and was found to be associated with AD by a hippocampal TWAS study (57). Pleiotropic SNP rs10774624 and locus 17 in general, are shared by phenotypes such as CAD (24), rheumatoid arthritis (58), systolic blood pressure (59), parental longevity (60) and various hematological traits (61). It was mapped to *ALDH2* by eQTL mapping approach in GTEx Whole Blood, brain nucleus accumbens basal ganglia and artery aorta tissues whose inactivating mutation has been linked to chronic excessive ethanol intake as potential contributors to Alzheimer's disease progression (58). Finally, *DNM2* intronic SNP rs2043332 has been linked to CAD (C allele: β = 0.04, *P* = 2.63e-09) (24) verifying ASSET's ability to discover novel trait associations. *DNM2*, as shown by the enrichment analysis in biological and functional categories, is involved in endocytosis (*via* the clathrin coated endocytic vesicle pathway) which is closely linked to the development of both Aβ and tau pathologies (62). A study in Japanese population suggested that *DNM2* via its rs892086 is a susceptibility gene for AD in non-APOE-ε4 carriers (63).

Interestingly, rs10818576-G, rs10774624-G, and rs1964272-G were associated with higher odds of developing CAD and lower odds of getting AD and surviving past 90 years old, whereas rs9630903-G was found to be protective of CAD but increasing the odds of developing AD and living longer. These directions of effects seem unexpected, however a previous study (10) reported ten similar variants whose unexpected direction of effect was replicated for only two of the SNPs. The authors suggested that these effects could be the result of interactions among variants, known as epistasis. Similar directions of effect have been reported in a study by Dato et al. (64) in which a SNP in *IP6K3* increased both lifespan and the risk of late-onset AD, while SNPs in *IPMK* and *UCP4* genes were associated with a lower risk of both late-onset AD and shorter lifespan. Thus, it is possible that SNP-SNP interactions may have different effects on AD and longevity depending on the genetic architecture of these traits and their associated underlying pathways. An alternative explanation could be that these SNPs have an age-dependent effect on traits; for example, hypertension at midlife increases the risk of AD, but after the age of 85 high blood is protective of AD (65).

The subset-based analysis also revealed variants that have pairwise, opposite shared effects. Eighteen lead SNPs across seven risk loci have potentially pleiotropic affects in both AD and longevity. *SPI1* (rs67472071, locus 14) is involved in the regulation of many genes, including the aging-related gene *WRN* (66). Various non-synonymous WRN coding region SNPs have been associated with age-related pathologies in different ethnic populations. For example, Kulminski and Culminskaya (67) reported that subjects with one A allele at the *WRN* SNP 1133A were associated with an earlier onset of cardiovascular diseases and cancer (and thus decreased longevity) when compared with individuals homozygous for a serine residue (S/S) at this SNP. *HBEGF* was found to be genome-wide significant risk gene for AD in a trans-ethnic study (44), is involved in Aβ clearance (68) and it has been reported to play a role in the increase of neurogenesis in the adult rat brain. Although neurogenesis persists in the aged brain, its rate declines with age in both rats and humans. Decreased hippocampal neurogenesis may be involved in age-related cognitive deficits because of its proposed role in learning and memory function (69). In addition, SNP rs11168036 (locus 6) was found to be associated with the expression of the *PCDHA* genes (Supplementary Table S7). The 5q31.3 region, which includes the genes *PCDHA1-PCDHA10*, influences the expression of the *WRD55* and *ARL4A* genes in the brain tissue (70). *ARL4A* have previously been associated with maternal longevity in an epigenome-wide association study of age and age-related phenotypes (71) indicating a possible link between the 5q31.3 deletion and longevity. Finally, rs9787911 has been found to be nominally associated with AD in the Japanese population (72). The mapped gene *NTM* at 11q25 encodes a protein that may promote neurite outgrowth and adhesion *via* a hemophilic mechanism (73) and a linkage at 11q25 for AD has been discovered by two studies (74, 75).

Among the variants that found by ASSET to be shared between CAD and longevity, *ING1* (rs9583531, locus 18) encodes a tumor suppressor protein that can induce cell growth arrest and apoptosis, is responsible for implementation of senescence and the reduced expression and rearrangement of this gene have been detected in various cancers (76). Recent evidence suggests that cellular senescence plays a regulatory role in the aging process (77). Furthermore, another of the pleiotropic genes, *IL6R* (rs4845619, locus 1), found to be associated with CAD in a later GWAS (24). It is involved in the pathophysiology of several age-related diseases such as osteoporosis (78) and reduced IL6 signaling lowers the risk of multiple cardiovascular disorders and is associated with increased longevity (79).

Finally, two SNPs showed pleiotropic effects among AD and CAD, namely rs116426890-T (locus 4) and rs62118504-G (locus 21). Locus 4 shows pleiotropic effects on various cardiovascular traits and apolipoprotein B levels as seen in Supplementary Table S5. SNP rs116426890 is intronic to *ABI2* and is linked to the expression of *CARF, ICA1L, FAM117B*, and *NBEAL1* (Supplementary Table S7) which are associated with both white matter hyperintensities and fractional anisotropy, predictors of cerebral small vessel disease which is involved in strokes and vascular dementia (80). Regarding *ABI2*, it is part of the same family as *ABI3* which is associated with AD (81). *ABI2's* protein has been found to be dysregulated in the entorhinal cortex at different stages of neurofibrillary tangle pathology compared with middle-aged individual and contributes to the regulation of actin assembly at the tips of neuron projections (82). SNP rs62118504-G, which is associated with AD based on the input AD GWAS, mapped to *EXOC3L2/MARK4* genes. The whole locus 21 is pleiotropic for various cardiovascular risk factors making it a strong candidate for being a CAD-associated loci as indicated by our cross-phenotype meta-analysis.

We acknowledge the limitations of our study. One limitation is the possibility of survival bias. Individuals predisposed to developing an age-related disease may not have survived to old age, which may have underestimated any association with these diseases. Moreover, the diagnosis of probable AD excludes a prior history of cerebrovascular disease, leading a reduced risk of overlap between the two diseases. It is also plausible that individuals reaching an age over 90 yeas carry a different genetic background to the general population consisting of protective SNPs that predispose for extreme longevity, which are not captured by our study. Furthermore, the input GWASs were of different sample sizes and power. Although the effects of such differences on the results are not fully understood, ASSET is known to be the best available approach to handling non-uniform distribution of sample sizes. The limitations of the ASSET approach include that its two-sided model can show a low accuracy due to the extensive search for subsets in both directions and thus, could lead to the identification of more false positive results when the significant associations are all in the same direction. However, in this study due to the negative genetic correlation among all phenotypes involved in the analysis, we expect associations to exist in opposite directions (as seen) which improves the power to identify the correct subset of traits. Moreover, the CARDioGRAMplusC4D contains some non-European participants but no heterogeneity among the studies was observed at any of the genome-wide significant variants apart from 9p21 locus. Finally, the only tissues which we used for mapping SNPs to genes, were those relevant to AD and CAD by showing a significant eQTL association, which may limit the number of mapped genes.

In summary, performing an association analysis by subsets across AD, longevity, and CAD we discovered novel (potentially) pleiotropic loci, and identify loci which were reported for individual-trait risk in later and larger GWAS studies. The latter suggests that increasing GWAS sample sizes is likely to identify more pleiotropic loci which are risk genes for more than one disease. Further work will be required to fully explore the role of aging in age-related diseases.

## Data availability statement

The datasets presented in this study can be found in online repositories. The names of the repositories can be found in the Supplementary material.

## Ethics statement

Ethical review and approval was not required as the datasets are openly accessible. The patients/participants provided their written informed consent to participate in those studies.

## Author contributions

EB: conceptualization, methodology, software, formal analysis, resources, writing - original draft, and visualization. VE-P: conceptualization, investigation, writing - review & editing, supervision, project administration, and funding acquisition. All authors contributed to the article and approved the submitted version.
