# Supplementary material for: Are Alzheimer's and coronary artery diseases genetically related to longevity?
Source: Front Psychiatry. 2023 Jan 6;13:1102347. doi: 10.3389/fpsyt.2022.1102347 (PMC9859055; doi:10.3389/fpsyt.2022.1102347)
Supplement: Supplementary file 1 [file Data_Sheet_1.docx]

Supplementary Material

# Supplementary Figures


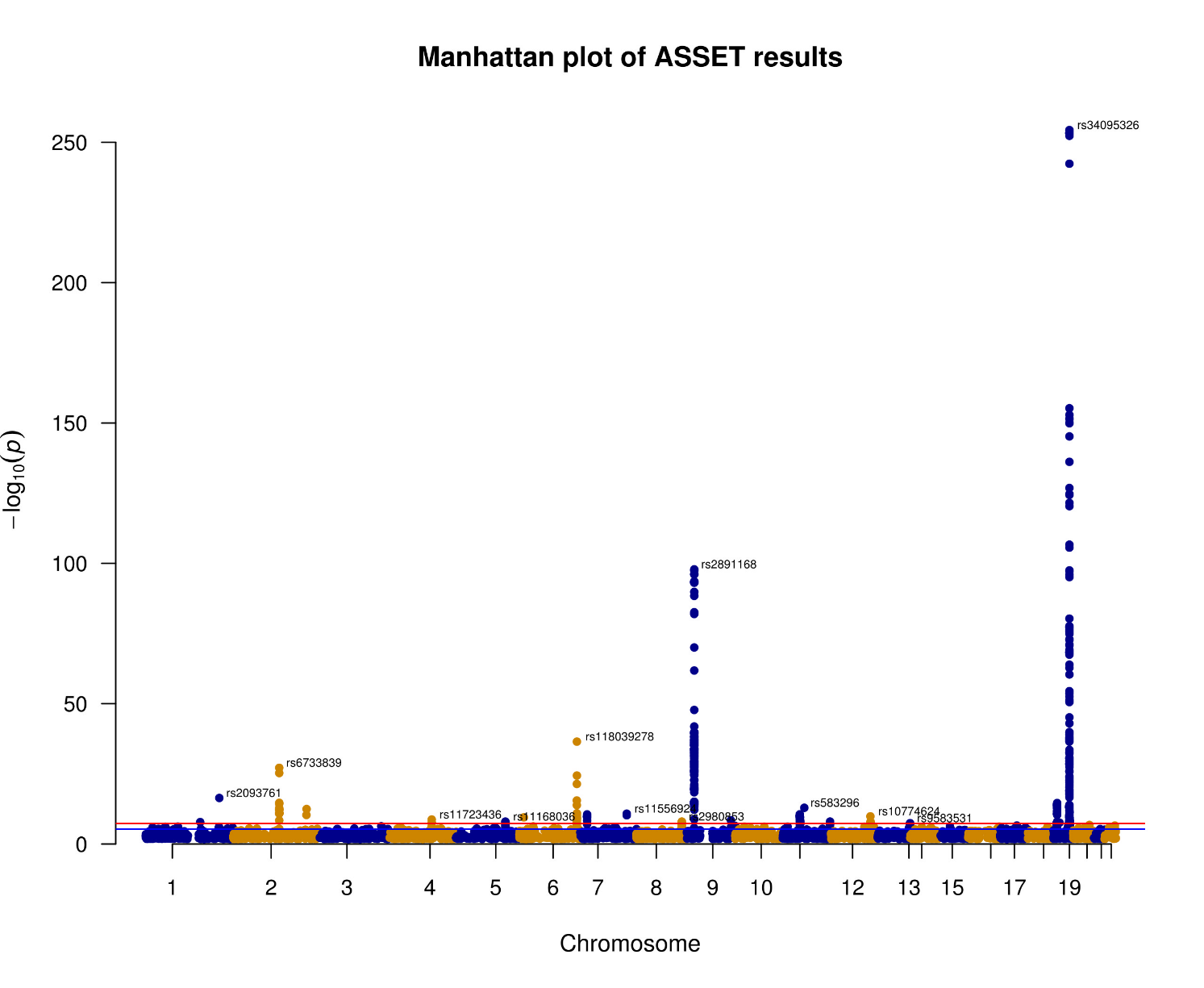


**Supplementary Figure 1.** Manhattan plot of the subset-based meta-analysis (ASSET) results. The red, horizontal line represents the genome-wide significance threshold 5e-08 and the blue the suggestive significance threshold of 5e-06. The top SNP per chromosome that exceeds the significance threshold of 5e-08 are depicted in the plot.

# Supplementary Tables

# The supplementary tables S1-S12 can be found in the provided excel file.
